# Clinical Mass Spectrometry Discovered Human IgG Sialylation as a Potential Biosignature for Kidney Function

**DOI:** 10.3390/jpm11080761

**Published:** 2021-07-31

**Authors:** Chih-Chin Kao, San-Yuan Wang, Yung-Kun Chuang, Wei-Yuan Lee, Wei-Chiao Chang, Mai-Szu Wu, Tai-Chih Kuo, I-Lin Tsai

**Affiliations:** 1 Division of Nephrology, Department of Internal Medicine, Taipei Medical University Hospital, Taipei 11031, Taiwan; d118102008@tmu.edu.tw (C.-C.K.); asia121711@gmail.com (W.-Y.L.); 2 Division of Nephrology, Department of Internal Medicine, School of Medicine, College of Medicine, Taipei Medical University, Taipei 11031, Taiwan; maiszuwu@tmu.edu.tw; 3 Taipei Medical University-Research Center of Urology and Kidney (TMU-RCUK), Taipei Medical University, Taipei 11031, Taiwan; 4 Master Program in Clinical Pharmacogenomics and Pharmacoproteomics, School of Pharmacy, Taipei Medical University, Taipei 11031, Taiwan; syw@tmu.edu.tw (S.-Y.W.); wcc@tmu.edu.tw (W.-C.C.); 5 Master Program in Food Safety, Taipei Medical University, Taipei 11031, Taiwan; ykchuang@tmu.edu.tw; 6 School of Food Safety, Taipei Medical University, Taipei 11031, Taiwan; 7 Nutrition Research Center, Taipei Medical University Hospital, Taipei 11031, Taiwan; 8 Department of Clinical Pharmacy, School of Pharmacy, Taipei Medical University, Taipei 11031, Taiwan; 9 Division of Nephrology, Department of Internal Medicine, Shuang Ho Hospital, Taipei Medical University, New Taipei City 23561, Taiwan; 10 Department of Biochemistry and Molecular Cell Biology, School of Medicine, College of Medicine, Taipei Medical University, Taipei 11031, Taiwan; tckuo@tmu.edu.tw; 11 Graduate Institute of Medical Sciences, College of Medicine, Taipei Medical University, Taipei 11031, Taiwan; 12 International PhD Program for Cell Therapy and Regeneration Medicine, College of Medicine, Taipei Medical University, Taipei 11031, Taiwan; 13 Pulmonary Research Center, Wan Fang Hospital, Taipei Medical University, Taipei 11031, Taiwan

**Keywords:** IgG, glycosylation, kidney function, mass spectrometry, estimated glomerular filtration rates

## Abstract

Immunoglobulin G (IgG) N-glycosylation was discovered to have an association with inflammation status, which has the potential to be a novel biomarker for kidney diseases. In this study, we applied an ultra-high performance liquid chromatography–tandem mass spectrometry (UHPLC-MS/MS) method to plasma and urine samples from 57 individuals with different levels of kidney function. Natural abundances of total IgG, IgG1, IgG2, and IgG3 subclasses in plasma showed positive correlations to the estimated glomerular filtration rates (eGFRs). Eighteen IgG glycopeptides also showed positive correlations. In contrast, higher IgG amounts were found in urine samples from participants with lower eGFR values. After normalizing IgG glycopeptides from plasma to their respective protein amounts, H4N4F1S1-IgG1 (*r* = 0.37, *p* = 0.0047, significant) and H5N4F1S1-IgG1 (*r* = 0.25, *p* = 0.063, marginally significant) were the two glycopeptides that still had positive correlations with eGFRs. The results showed that the UHPLC-MS/MS method is capable of investigating IgG profiles, and monitoring IgG and glycosylation patterns is worthy of further clinical application for kidney disease.

## 1. Introduction

The global burden of chronic kidney disease (CKD) is substantial in terms of both economic and disease impacts [1,2]. CKD contributes to an increased risk of cardiovascular disease mortality, independent of other comorbidities [3], and it is reported to have a prevalence of 3~17% worldwide [4,5,6,7]. CKD is characterized by reduced kidney function and structural kidney damage lasting at least 3 months [8]. It arises from heterogeneous diseases, but diabetes and hypertension are the two main causes [9]. Some other causes of CKD include glomerulonephritis, viral infections, side effects of herbal medicines, and unknown causes. The common pathological manifestation of CKD is renal fibrosis, characterized by glomerulosclerosis, tubular atrophy, and interstitial fibrosis. Micro-inflammation is the culprit mechanism that contributes to CKD, as evidenced by inflammatory cell infiltration and immunoglobulin (Ig) deposition in most renal biopsies.

A proportion of CKD patients develop end-stage renal disease (ESRD) and require long-term renal replacement therapy; however, it remains difficult to predict patients who will progress to ESRD. Some groups have tried to determine specific markers to predict kidney function progression by proteomics investigation [10]. Sets of 13 upregulated and 14 downregulated glycoproteins were found in the plasma of diabetic nephropathy patients [11]. The urine glycoproteomic profile has also been reported to be a biomarker for the early detection and prognosis of CKD in several studies [12,13]. However, the results of those studies were heterogeneous and inconsistent. A distinct manifestation of the immunoglobulin (Ig) level related to kidney injury was found in patients with a plasma cell disorder. Paraproteinemia is associated with kidney injury, either by excessive monoclonal Ig deposition, amyloid deposition, or light chain proximal tubulopathy [14]. Therefore, we aimed to investigate the IgG profiles in terms of natual abundances and glycosylation patterns, which have the potential to be correlated with kidney function.

Human IgGs are classified into four subclasses, and the glycosylation profiles of their fragment crystallizable region (Fc region, Asn 297) are associated with proinflammatory and anti-inflammatory statuses. Different glycosylation patterns of the IgG subclasses affect their stability and affinity to IgG receptors or complements. Many studies have used mass spectrometry (MS)-based analytical methods to monitor IgG profiles for biomarker discovery and disease diagnosis [15,16]. For CKD, a few studies have investigated the correlations of IgG glycosylation or the IgGs level to kidney function [17]. However, different techniques were used in these studies to determine the biomolecular targets. The advantages of using liquid chromatography-mass spectrometry include sensitivy, specificity, and multitarget analysis. Moreover, a validated method with a suitable quantitative range could provide accurate results with relative quantification for analytes. In this study, we applied an accurate and validated LC-MS/MS method to investigate IgG subclasses and glycosylation profiles in plasma and urine samples. The profiles were correlated to eGFR values, to identify potential glycopeptides as an indicator of kidney function.

## 2. Materials and Methods

### 2.1. Reagents and Standards

Natural human IgG standards, including IgG1, IgG2, IgG3, and IgG4, were purchased from Abcam (Cambridge, MA, USA). The internal standard used in this study was the SILu™MAB K1 Stable-Isotope-Labeled Universal Monoclonal Antibody, purchased from Sigma-Aldrich (St. Louis, MO, USA). Iodoacetamide (IAA), ammonium bicarbonate, and formic acid were also purchased from Sigma-Aldrich. Dithiothreitol (DTT) was purchased from Merck (Billerica, MA, USA). Protein G Mag Sepharose Xtra beads were obtained from GE (Piscataway, NJ, USA). Chicken serum, used as a blank matrix, was purchased from Thermo Scientific (Sunnyvale, CA, USA). Phosphate-buffered saline (PBS; 10×, sterile solution) was purchased from VWR International (West Chester, PA, USA). Lyophilized trypsin was purchased from Promega (Madison, WI, USA). MS-grade acetonitrile (ACN) was purchased from J.T. Baker (Phillipsburg, NJ, USA).

### 2.2. IgG Purification and Ultrahigh Performance Liquid Chromatographic (UHPLC)–Tandem MS (MS/MS) Analysis

Sample preparation and analytical conditions were previously developed and validated by our team [18]. Briefly, 60 μL of magnetic bead slurry immobilized with protein G was used for IgG purification. PBS was used for bead conditioning, and 2 μL of human plasma was added to a 200 μL microtube for IgG purification. For urine IgG purification, 900 μL of urine was added into a 1.5 mL microtube with preconditioned protein G magnetic beads. The K1 internal standard (10 μL, 0.2 μg/μL) was added to each sample before initiating the purification step. Incubation was conducted at room temperature for 30 min. After washing, on-bead protein digestion was used to obtain tryptic peptides and glycopeptides of IgG. Ammonium bicarbonate (50 μL, 50 mM) was added to the microtube with washed beads, and 1 μL of 550 mM DTT was added for disulfide bond reduction (at 56 °C for 45 min). Two microliters of IAA (450 mM) were added for cysteine alkylation (45 min in the dark). Trypsin (10 μL, 200 ng/μL) was spiked into each sample for protein digestion (16 h at 200 rpm). To stop the protein digestion process, 6 μL of 10% formic acid was added to acidify the sample solution. The supernatant with tryptic peptides and glycopeptides was collected after centrifuging samples at 12,000 rpm for 15 min, and the supernatant was diluted with 0.1% formic acid four times prior to the UHPLC-MS/MS analysis.

The Waters ACQUITY UPLC system (quaternary pumps) coupled with an Xevo TQ-XS triple quadruple mass spectrometry system (Waters, Milford, MA, USA) was used for sample analysis. A Kinetex^®^ Core-Shell C18 (50 × 2.1 mm, 2.6 µm; Phenomenex, Torrance, CA, USA) column was used in this study for analyte separation. Dynamic multiple reaction monitoring (MRM) was applied to target peptides representing total IgG, four IgG subclasses, and 26 IgG glycopeptides. Information on the target analytes is summarized in Supplemental Table S1.

### 2.3. Clinical Sample Collection and Application

In this study, we analyzed IgG and IgG glycopeptides in plasma and urine samples from 57 participants with CKD, and healthy controls. Participants were enrolled in Taipei Medical University Hospital, and the study was approved by the ethics committee (N201704064). Informed consent forms were provided, and signed forms were received from all participants. Whole blood (10 mL) was collected, and the plasma fraction was obtained by centrifuging the blood at 3000 relative centrifugal force (RCF) for 15 min. A urine sample was also collected, and sodium azide was added to a final concentration of 10 mM as preservative. All plasma and urine samples were stored in a −80 °C freezer before use.

### 2.4. ELISA Assays for Interleukin-6 (IL-6), Tumor Necrosis Factor-α (TNF-α), and Creatinine

Plasma concentrations of interleukin-6 (IL-6) and tumor necrosis factor-α (TNF-α) were determined by using ELISA kits: Human IL-6 ELISA Kit, RAB0306-1KT, LOT#: 0428I140; Human Tumor Necrosis Factor alpha ELISA Kit, RAB0476-1LT, Lot#: 0422I193 (Sigma-Aldrich, St. Louis, MO, USA). Brifly, calibration curves were prepared by using protein standards with suitable serial dilutions. Fifty microliters of plasma were diluted with buffer solution two times for sample incubation. After adding antibodies and reporter reagent for a specified incubation time, the signals were determined with a microplate spetrophotometer (450 nm). The optical density (OD) values were used to calculate the analyte concentrations. Urine creatinine concentrations were also determined by using a commercially available ELISA kit: Parameter^TM^ Creatinine Assay, KGE005, LOT#: P289536 (R&D Systems, Minneapolis, MN, USA). Ten microliters of urine were diluted with buffer solution 20 times for sample incubation. After adding alkaline picrate solution for a 30 min incubation time, the signals were determined with a microplate spetrophotometer (490 nm).

### 2.5. Data Analysis and Statistical Analysis

All raw UHPLC-MS/MS data were processed by MassLynx (vers. 4.2, Waters, Milford, MA, USA). The peak area and peak area ratio (responses) of all signals were integrated and calculated by the software, and exported to Excel files for further analysis. Natural abundance (Response) indicated in this study was the peak area of the analyte divided by the peak area of the internal standard. The normalized area indicated in the study was the natural abundance of the analytes divided by the abundance of their respective proteins. Correlations between IgG profiles and the estimated glomerular filtration rate (eGFR) were calculated by GraphPad Prism (vers. 5.01, San Diego, CA, USA). Statistical analysis of glycopeptides and cytokines between two or four groups defined in this study were conducted by Mann-Whitney test and Kruskal Wallis test, respectively (GraphPad Prism).

## 3. Results

### 3.1. Clinical Participants

To investigate the relationships between IgG profiles and kidney function, 22 female and 35 male participants were recruited for this study. The ages ranged 27~77 years old, and the eGFR values ranged 15~171 mL/min/m^2^. In Table 1, we grouped participants into four categories based on their eGFR values. The ages of participants showed no significant differences among the four groups, but there were more males with lower kidney function. The detailed characteristics of the 57 participants are summarized in Supplemental Table S2.

### 3.2. Performance of the Analytical Platform

**Figure 1:**
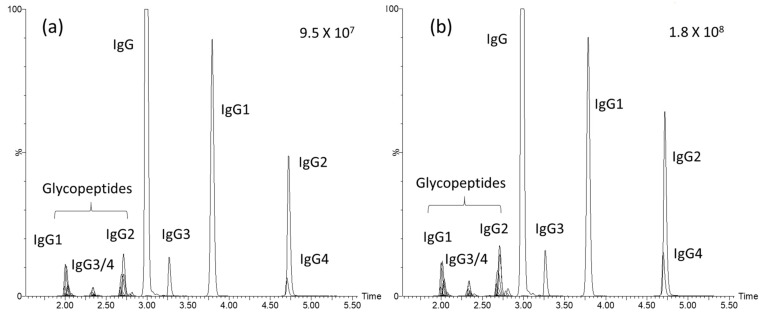
Chromatograms of total immunoglobulin G (IgG), IgG subclasses, and IgG glycopeptides from individuals with low and high eGFR values. (**a**) Participant with an eGFR of 15 mL/min/1.73 m^2^. (**b**) Participant with an eGFR of 171 mL/min/1.73 m^2^.

UHPLC-MS/MS is one type of analytical platform, with the advantages of high sensitivity and selectivity, with which it is possible to determine multiple analytes in one injection. It has been widely applied to biological and clinical samples for small molecule and biomolecule analyses. IgG and glycopeptides have recently been monitored and correlated with many disease statuses as biological signatures for diagnosis or progression [19,20]. In those studies, LC coupled with MS was usually the main analytical tool. In our previous work, we also developed and validated a UHPLC-MS/MS method to achieve absolute quantification of the four IgG subclasses, and relative quantification of 26 IgG glycopeptides, from plasma samples [18]. In this study, we applied the same workflow, to which a stable isotope-labeled IgG internal standard was added to each sample. The calibration curve for each target was generated to confirm the reliability of comparing the responses (peak area ratio) of analytes among samples. The performances of the calibration curves for the target analytes are summarized in Supplemental Table S1. Values of the correlation coefficients (*r*) of the calibration curves for total IgG, IgG subclasses, and the 26 glycopeptides ranged from 0.947~0.999. The accuracies of the QC samples at four concentrations ranged from 83.4%~103.5% for total IgG and the IgG subclasses. Total ion current chromatograms from two individuals with high and low eGFR values are shown in Figure 1.

### 3.3. Correlations of Natural Abundances of IgG Profiles and Glycopeptides to the eGFR

**Figure 2:**
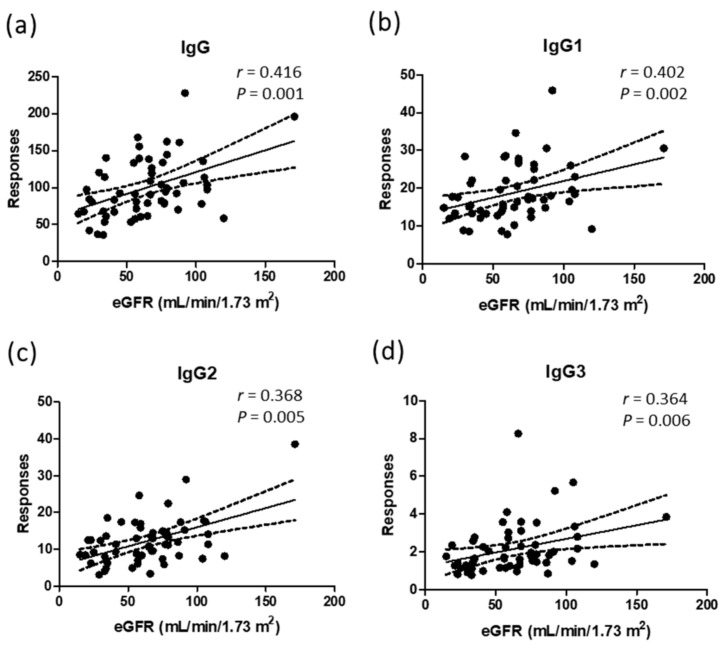
Correlations of natural abundances of total IgG and IgG subclasses to the values of eGFR from 56 participants. (**a**) Total IgG; (**b**) subclass IgG1; (**c**) subclass IgG2; (**d**) subclass IgG3. *r* is Spearman’s *r*. *p* represents the *p*-value from correlation analysis. The peak area of each target was divided by the peak area of the internal standard, DSTYSLSSTLTLSK.

After analyzing total IgG, IgG subclasses, and the 26 glycopeptides from the plasma of 57 participants, we found that 1 patient had a high abundance of IgG. Although the patient did not have a pathological history of infection or autoimmune disease, there was a clinical issue of knee osteoarthritis, which might have been the reason for the high plasma IgG level. Therefore, we excluded the experimental results from this patient to prevent bias. From the results of 56 participants, we found that the natural abundances of total IgG, IgG1, IgG2, and IgG3 were positively correlated with eGFR values. The *r* values ranged from 0.36~0.42. All *p* values were <0.05. Only human IgG4 did not show a correlation with kidney function indicators (Figure 2).

**Figure 3:**
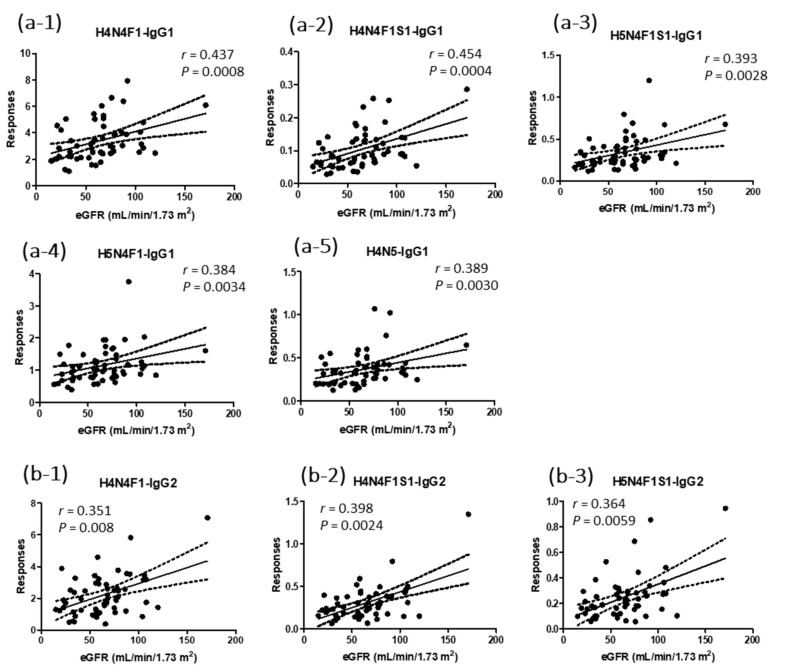
Correlations of natural abundances of IgG1 and IgG2 N-glycopeptides to the values of eGFR from 56 participants. (**a**) IgG1 glycopeptides with *p* values less than 0.01, (**b**) IgG2 glycopeptides with *p* values less than 0.01. *r* is Spearman’s *r*. The peak area of each target was divided by the peak area of the internal standard, DSTYSLSSTLTLSK.

**Figure 4:**
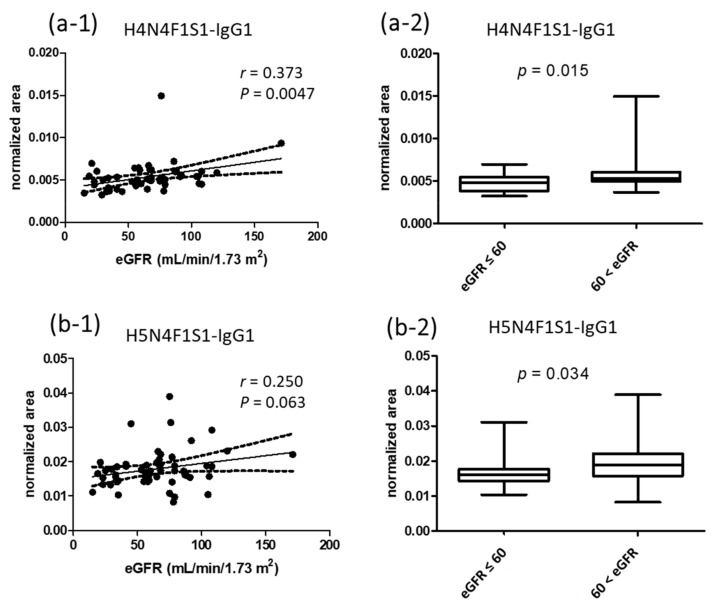
Correlations of the normalized area of H4N4F1S1-IgG1 and H5N4F1S1-IgG1 to the values of eGFR from 56 participants (**a**-**1**,**b**-**1**). Statistical analysis of the normalized area of H4N4F1S1-IgG1 and H5N4F1S1-IgG1 between groups with eGFR values lower and higher than 60 mL/min/1.73 m^2^ (**a**-**2**,**b**-**2**). *r* is Spearman’s *r*. The peak area of each target was normalized to the peak area of the internal standard, DSTYSLSSTLTLSK, and also the responses of IgG1.

**Figure 5:**
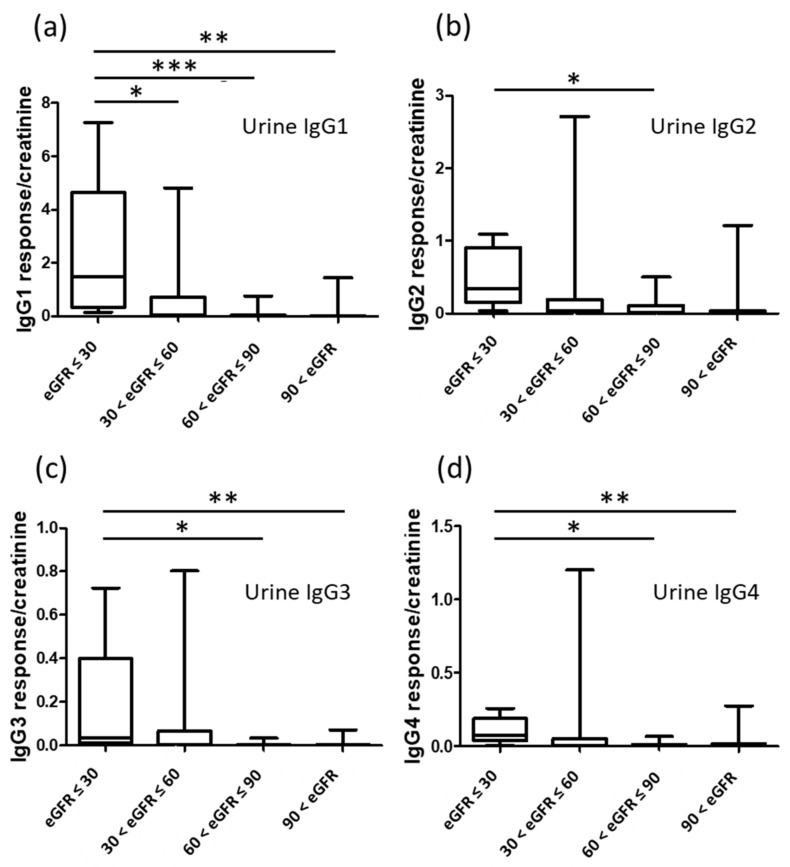
Comparison of the amount of urine IgG subclasses between the four groups with different eGFR values. (**a**) IgG1, (**b**) IgG2, (**c**) IgG3, (**d**) IgG4. *p* values represent statistically significant differences: * *p* < 0.05, ** *p* < 0.01, *** *p* < 0.001.

In this study, we also analyzed 26 N-glycopeptides of IgG. As for the natural protein levels in plasma, 18 N-glycopeptides from IgG1 and IgG2 showed positive correlations with eGFR values (Figure 3). The *r* values for the eight IgG1 glycopeptides ranged from 0.27~0.45. H4N4F1-IgG1 and H4N4F1S1-IgG1 showed *p* values < 0.001 (Figure 3(a-1,a-2)). H5N4F1-IgG1, H5N4F1S1-IgG1, and H4N5-IgG1 were the three IgG1 glycopeptides with *p* values < 0.01 (Figure 3(a-3–a-5)). The *r* values for the 10 IgG2 glycopeptides ranged from 0.32~0.40. H4N4F1-IgG2, H4N4F1S1-IgG2, and H5N4F1S1-IgG2 showed *p* values of <0.01 (Figure 3(b-1–b-3)). Glycopeptides of IgG3/4 showed no correlations with the eGFR values. The *r* and *p* values for the 26 glycopeptides are summarized in Supplemental Table S3.

### 3.4. Correlations of Normalized Signals of Glycopeptides to eGFR Values

Since the natural abundances of glycopeptides are mainly affected by their protein levels, we further normalized the abundances of glycopeptides to their respective protein amounts. For example, the responses of IgG1 glycopeptides were divided by the response of IgG1 peptide. After normalization, normalized signals were used to investigate their correlations to eGFR values. Among the 26 glycopeptides, only H4N4F1S1-IgG1 and H5N4F1S1-IgG1 showed *p* values smaller than and approaching 0.05 (Figure 4(a-1,b-1)). We then analyzed the normalized area of the two glycopeptides among the four eGFR groups, but there were no significant differences. To increase the n numbers for statistics, we classified the participants into two groups, with eGFR values lower and higher than 60 mL/min/1.73 m^2^. The results showed that there were statistically significant differences between the groups for the two sialylated glycopeptides (Figure 4(a-2,b-2)).

### 3.5. Natural Abundances of IgG Subclasses in Urine Samples

Urine samples from 56 participants were processed with the same IgG purification method, and the purified IgG was analyzed by the developed UHPLC-MS/MS method. The signals of the glycopeptides were quite small compared to those from the plasma samples. Therefore, we only analyzed signals of IgG subclass representative peptides, and the natural abundances (responses) were further normalized with the urine creatinine concentration to account for the variation of the urine flow rate. The creatinine normalized urine IgG subclasses were used for statistical analysis. In Figure 5, the amount of urine IgGs was higher in the groups with lower eGFR values.

### 3.6. Quantification Results of IL-6 and TNF-α

Plasma cytokines related to inflammation were determined by using ELISA assay kits. The concentrations of both Il-6 and TNF-α showed no statistical differences between the four groups with different levels of eGFR values. We further compared the cytokine levels between two groups, with eGFR levels lower or higher than 60 mL/min/1.73 m^2^. There were still no significant differences between the two groups (Supplemental Figure S1).

## 4. Discussion

To achieve accurate quantitative analyses of biological signatures, it is necessary to use a reliable analytical method to determine features from complicated bio-fluids. In this study, we used a UHPLC-MS/MS method which was previously developed and validated by our team. A stable isotope-labeled internal standard was added prior to IgG purification to calibrate variations due to sample preparation and analysis. Calibration curves were also generated for total IgG, IgG subclasses, and glycopeptides, to confirm the accuracy of responses for further correlation analyses.

From the study results, we found that the plasma levels of total IgG, IgG1, IgG2, and IgG3 subclasses decreased among individuals with lower eGFR values. Dr. Yen and team members have reported that decreased serum IgG and creatinine have the potential to be predictors for non-diabetic renal disease (NDRD) in patients with type 2 diabetes mellitus [21]. However, they also pointed out that increased IgG and glycosylation were associated with the onset of early type 1 diabetic nephropathies. Although we did not categorize patients according to their pathological histories, our hypothesis is that individuals with lower eGFR values might have more macromolecular excretion in the urine, including IgG. Higher excretion might result in lower serum levels of IgG and its subclasses. This hypothesis was supported by the results of our analysis of urine samples; higher amounts of IgG were discovered in urine samples from participants with lower eGFR values. Detailed classification of disease groups and comparisons with other clinical data can be conducted in the future.

Since different glycosylation levels of the IgG Fc region have been reported to have proinflammatory or anti-inflammatory effects, studies of N-glycosylation of IgG have been used in clinical applications, including kidney transplants, kidney function in type 2 diabetes, and moderate kidney dysfunction [17,22,23]. In a study published by Dr. Barrios and Dr. Menni et al., they found 14 IgG glycan traits belonging to galactosylation, sialylation, and bisecting features, which were associated with kidney function [17]. In the current research results, we found that the IgG1 glycopeptide carrying sialic acid (H4N4F1S1-IgG1) showed a moderate correlation with eGFR values, even after normalizing the natural abundances to the respective protein level (*r* = 0.37, *p* = 0.0047). Another glycopeptide, H5N4F1S1-IgG1, with sialic acid, also showed a moderate correlation, but with a relatively higher *p* value. Compared to human IgA isotype, IgG is less sialylated, and sialylation of the IgG Fc region decreases their affinities to the Fcγ receptor, which was pointed out to be anti-inflammatory. Our finding corresponds to the results from Drs. Bassios and Menni et al., in which a decrease in sialylation was found in patients with CKD [17]. The decrease of sialylated IgG revealed the inflammatory status of individuals with lower kidney function. This assumption was supported by the study results reported by Dr. Chen et al., in which higher inflammation cytokines, such as IL-6 and TNF-α, were found in patients with lower eGFR [24]. Although our study showed no significant association between IL-6, TNF-α and eGFR, the results might be due to many samples having cytokine concentrations lower than the quantification limits of the ELISA kits, which affected the statistics.

Age and gender are two important factors which also affect IgG N-glycosylation profiles. In our study, the ages of participants showed no significant differences among the four eGFR groups, but more males had lower eGFR values. Therefore, we checked the literature to see if monosialylation (H4N4F1S1-IgG1 and H5N4F1S1-IgG1) was gender-related. A study published by Dr. Gornik et al. found that there were no significant difference of sialylation of IgG in boys and girls (<18 years) [25]. A study published by Dr. Li et al. found that galactosylation, but not sialylation, was more gender-related [26]. Based on these results, we believe that H4N4F1S1-IgG1 and H5N4F1S1-IgG1 have the potential to be biosignatures for kidney function.

There are still limitations of the study. We assumed that renal damage plays a primary role in modulating IgG plasma levels based on the results of reduction in plasma IgG and the concomitant increase in urinary IgG at lower eGFR levels. However, the pathological histories for the current population are heterogeneous which made detailed classification difficult. It is hard for us to correlate discovered IgG profiles to different types of renal damages at the current stage. The correlations of sialylated IgG peptides to the pathogenesis and the progression of renal damage also require further investigation.

## 5. Conclusions

Kidney function is affected by many biological factors and diseases. Novel biosignatures for predicting and monitoring kidney functions need to be discovered. Inflammation is known to be involved in the progression of nephropathies, and IgG glycosylation has shown correlations with different kidney diseases. In this study, we applied a validated UHPLC-MS/MS method to determine IgG glycosylation profiles from individuals with different eGFR values. The natural abundances of total IgG, IgG1, IgG2, IgG3, and 18 IgG N-glycopeptides showed lower levels in patients with lower eGFR values. After normalizing the responses of glycopeptides to their respective proteins, two glycopeptides with sialic acid, H4N4F1S1-IgG1 and H5N4F1S1-IgG1, showed positive correlations with eGFR values, which were statistically significant and marginally significant, respectively. The results corresponded to previous findings that lower blood levels of IgG and less sialylation occur in patients with kidney diseases. The detailed pathological mechanism between IgG and kidney function is worthy of further investigation.

## Figures and Tables

**Table 1:** The four groups of participants with different eGFR values.

eGFR (mL/min/1.73 m^2^)	eGFR ≤ 30	30 < eGFR ≤ 60	60 < eGFR ≤ 90	90 < eGFR
Number of participants	8	19	21	9
Gender	Male: 8	Male: 14; Female: 5	Male: 10; Female: 11	Male: 3; Female: 6
Age (years old)	56.5 ± 13.7	59.5 ± 11.9	62.5 ± 11.1	52.6 ± 16.7

## Data Availability

The data that have been used to support the findings of this study are available from the corresponding author upon request.

## Supplementary Materials

The following are available online at https://www.mdpi.com/article/10.3390/jpm11080761/s1, Table S1: Analytes monitored in the study by using UHPLC-MS/MS. Peptides represent total IgG, IgG1, IgG2, IgG3, and IgG4 were used to monitor the protein amounts in plasma. Correlation coefficients (r) of calibration curves are listed in the table, Table S2: Characteristics of 57 participants in the study, Table S3: Results of correlation analysis of natural abundances and normalized abundances of 26 IgG glycopeptides to the eGFR values; Figure S1: Statistical analysis of plasma concentrations of IL-6 (a) and TNF-α (b) between groups with eGFR values lower and higher than 60 mL/min/1.73 m^2^.

## References

[1] Levey A.S., Atkins R., Coresh J., Cohen E.P., Collins A.J., Eckardt K.U., Nahas M.E., Jaber B.L., Jadoul M., Levin A. (2007). Chronic kidney disease as a global public health problem: Approaches and initiatives—A position statement from Kidney Disease Improving Global Outcomes. Kidney Int..

[2] (2020). Global, regional, and national burden of chronic kidney disease, 1990–2017: A systematic analysis for the Global Burden of Disease Study 2017. Lancet.

[3] Sarnak M.J., Levey A.S., Schoolwerth A.C., Coresh J., Culleton B., Hamm L.L., McCullough P.A., Kasiske B.L., Kelepouris E., Klag M.J. (2003). Kidney disease as a risk factor for development of cardiovascular disease: A statement from the American Heart Association Councils on Kidney in Cardiovascular Disease, High Blood Pressure Research, Clinical Cardiology, and Epidemiology and Prevention. Circulation.

[4] Brück K., Stel V.S., Gambaro G., Hallan S., Völzke H., Ärnlöv J., Kastarinen M., Guessous I., Vinhas J., Stengel B. (2016). CKD Prevalence Varies across the European General Population. J. Am. Soc. Nephrol. Jasn.

[5] Stanifer J.W., Jing B., Tolan S., Helmke N., Mukerjee R., Naicker S., Patel U. (2014). The epidemiology of chronic kidney disease in sub-Saharan Africa: A systematic review and meta-analysis. Lancet Glob. Health.

[6] Wen C.P., Cheng T.Y., Tsai M.K., Chang Y.C., Chan H.T., Tsai S.P., Chiang P.H., Hsu C.C., Sung P.K., Hsu Y.H. (2008). All-cause mortality attributable to chronic kidney disease: A prospective cohort study based on 462 293 adults in Taiwan. Lancet.

[7] Coresh J., Selvin E., Stevens L.A., Manzi J., Kusek J.W., Eggers P., van Lente F., Levey A.S. (2007). Prevalence of chronic kidney disease in the United States. JAMA.

[8] Stevens P.E., Levin A. (2013). Evaluation and management of chronic kidney disease: Synopsis of the kidney disease: Improving global outcomes 2012 clinical practice guideline. Ann. Intern. Med..

[9] Webster A.C., Nagler E.V., Morton R.L., Masson P. (2017). Chronic Kidney Disease. Lancet.

[10] Klein J., Kavvadas P., Prakoura N., Karagianni F., Schanstra J.P., Bascands J.L., Charonis A. (2011). Renal fibrosis: Insight from proteomics in animal models and human disease. Proteomics.

[11] Ahn J.M., Kim B.G., Yu M.H., Lee I.K., Cho J.Y. (2010). Identification of diabetic nephropathy-selective proteins in human plasma by multi-lectin affinity chromatography and LC-MS/MS, Proteomics. Clin. Appl..

[12] Vivekanandan-Giri A., Slocum J.L., Buller C.L., Basrur V., Ju W., Pop-Busui R., Lubman D.M., Kretzler M., Pennathur S. (2011). Urine glycoprotein profile reveals novel markers for chronic kidney disease. Int. J. Proteom..

[13] Rossing K., Mischak H., Dakna M., Zürbig P., Novak J., Julian B.A., Good D.M., Coon J.J., Tarnow L., Rossing P. (2008). Urinary proteomics in diabetes and CKD. J. Am. Soc. Nephrol..

[14] Doshi M., Lahoti A., Danesh F.R., Batuman V., Sanders P.W. (2016). Paraprotein-Related Kidney Disease: Kidney Injury from Paraproteins-What Determines the Site of Injury?. Clin. J. Am. Soc. Nephrol..

[15] Chang Y.T., Chang M.C., Tsai Y.J., Ferng C., Shih H.C., Kuo Y.P., Chen C.H., Tsai I.L. (2019). Method development of immunoglobulin G purification from micro-volumes of human serum for untargeted and targeted proteomics-based antibody repertoire studies. J. Food Drug Anal..

[16] Gudelj I., Lauc G., Pezer M. (2018). Immunoglobulin G glycosylation in aging and diseases. Cell Immunol..

[17] Barrios C., Zierer J., Gudelj I., Štambuk J., Ugrina I., Rodríguez E., Soler M.J., Pavić T., Šimurina M., Keser T. (2016). Glycosylation Profile of IgG in Moderate Kidney Dysfunction. J. Am. Soc. Nephrol..

[18] Shiao J.Y., Chang Y.T., Chang M.C., Chen M.X., Liu L.W., Wang X.Y., Tsai Y.J., Kuo T.C., Tsai I.L. (2020). Development of efficient on-bead protein elution process coupled to ultra-high performance liquid chromatography-tandem mass spectrometry to determine immunoglobulin G subclass and glycosylation for discovery of bio-signatures in pancreatic disease. J. Chromatogr. A.

[19] Bhargava R., Lehoux S., Maeda K., Tsokos M.G., Krishfield S., Ellezian L.Y., Pollak M., Stillman I.E., Cummings R.D., Tsokos G.C. (2021). Aberrantly glycosylated IgG elicits pathogenic signaling in podocytes and signifies lupus nephritis. JCI Insight.

[20] Sołkiewicz K., Krotkiewski H., Jędryka M., Kratz E.M. (2021). Variability of serum IgG sialylation and galactosylation degree in women with advanced endometriosis. Sci. Rep..

[21] Weng C.H., Hu C.C., Yu C.C., Lin J.L., Yang C.W., Hung C.C., Hsu C.W., Yen T.H. (2012). Immunoglobulin G levels can predict non-diabetic renal disease in patients with type 2 diabetes mellitus. J. Diabetes.

[22] Bhargava R., Maeda K., Tsokos M.G., Pavlakis M., Stillman I.E., Tsokos G.C. (2021). N-glycosylated IgG in patients with kidney transplants increases calcium/calmodulin kinase IV in podocytes and causes injury. Am. J. Transplant..

[23] Singh S.S., Heijmans R., Meulen C.K.E., Lieverse A.G., Gornik O., Sijbrands E.J.G., Lauc G., van Hoek M. (2020). Association of the IgG N-glycome with the course of kidney function in type 2 diabetes. BMJ Open Diabetes Res. Care.

[24] Lee B.T., Ahmed F.A., Hamm L.L., Teran F.J., Chen C.S., Liu Y., Shah K., Rifai N., Batuman V., Simon E.E. (2015). Association of C-reactive protein, tumor necrosis factor-alpha, and interleukin-6 with chronic kidney disease. BMC Nephrol..

[25] Pucic M., Muzinic A., Novokmet M., Skledar M., Pivac N., Lauc G., Gornik O. (2012). Changes in plasma and IgG N-glycome during childhood and adolescence. Glycobiology.

[26] Chen G., Wang Y., Qiu L., Qin X., Liu H., Wang X., Wang Y., Song G., Li F., Guo Y. (2012). Human IgG Fc-glycosylation profiling reveals associations with age, sex, female sex hormones and thyroid cancer. J. Proteom..

