# Supplementary material for: Clinical Mass Spectrometry Discovered Human IgG Sialylation as a Potential Biosignature for Kidney Function
Source: J Pers Med. 2021 Jul 31;11(8):761. doi: 10.3390/jpm11080761 (PMC8401842; doi:10.3390/jpm11080761)
Supplement: Supplementary file 1 [file jpm-11-00761-s001.zip › jpm-1315812-SI.pdf]

Supplemental Table S1. Analytes monitored in the study by using UHPLC-MS/MS. Peptides represent total IgG, IgG1, IgG2, IgG3, and IgG4 were used to monitor the protein amounts in plasma. Correlation coefficients (*r*) of calibration curves are listed in the table.

**Analyte**	**Q1[a]**	**Q2**	***r***
(1) H4N4F1-IgG1	932.8	204.1	0.978
(2) H5N4F1-IgG1	986.8	366.1	0.978
(3) H3N4F1-IgG1	878.8	204.1	0.978
(4) H4N4-IgG1	884.1	204.1	0.975
(5) H5N4-IgG1	938.1	366.1	0.975
(6) H4N5F1-IgG1	1000.5	204.1	0.978
(7) H3N5F1-IgG1	946.5	204.1	0.973
(8) H5N5F1-IgG1	1054.5	366.1	0.976
(9) H4N5-IgG1	951.7	204.1	0.977
(10) H5N4F1S1-IgG1	1083.8	366.1	0.978
(11) H4N4F1S1-IgG1	1029.8	204.1	0.980
(12) H3N4F1-IgG3/4	873.4	204.1	0.997
(13) H4N4F1-IgG3/4	927.4	204.1	0.987
(14) H3N5F1-IgG3/4	941.1	204.1	0.981
(15) H4N5F1-IgG3/4	995.1	204.1	0.982
(16) H5N4F1S1-IgG3/4	1078.4	366.1	0.975
(17) H5N4F1-IgG2	976.1	366.1	0.990
(18) H5N5F1-IgG2	1043.8	366.1	0.987
(19) H4N4F1-IgG2	922.1	204.1	0.992
(20) H4N5F1-IgG2	989.9	204.1	0.991
(21) H4N4-IgG2	873.4	204.1	0.985
(22) H4N5-IgG2	941.1	204.1	0.947
(23) H3N4F1-IgG2	868.1	204.1	0.989
(24) H3N5F1-IgG2	935.8	204.1	0.988
(25) H5N4F1S1-IgG2	1073.1	366.1	0.979
(26) H4N4F1S1-IgG2	1019.1	204.1	0.980
IgG - DTLMISR	418.2	310.2	0.999
		506.3	
		619.4	
IgG1 - GPSVFPLAPSSK	593.8	418.2	0.998
		699.4	
		846.5	

IgG2 - GLPAPIEK	953.5	804.4	0.997
		852.4	
		1150.5	
IgG3 - WYVDGVEVHNAK	472.9	484.7	0.999
		534.3	
		697.4	
		853.5	
IgG4 - TTPPVLDSDGSFFLYSR	951.5	850.4	0.996
		1293.6	
DSTYSLSSTLTLSK (IS)	755.9	844.5	NA
		957.6	
		1044.6	

[a] Q1 represents parent ions and Q2 represents product ions.

[b] IgG1 glycopeptide: glycan-EEQYNSTYR; H, hexose; N, N-acetylglucosamine; F, fucose; S, N-acetylneuraminic acid; IgG2 glycopeptide: glycan-EEQFNSTFR; IgG3/4 glycopeptide: glycan-EEQYNSTFR (IgG3); glycan-EEQFNSTYR (IgG4)

Supplemental Table S2. Characteristics of 57 participants in the study.

Case number	Age	Gender	eGFR (mL/min/1.73m2)	Primary disease[a]
1	57	M	21	GN
2	29	M	86	GN
3	72	F	88	GN
4	62	M	55	GN
5	60	F	91	GN
6	68	F	67	HTN
7	60	F	78	GN
8	64	M	59	GN
9	72	F	75	GN
10	64	F	68	GN
11	71	M	30	DMN, HTN
12	68	M	71	GN
13	53	M	68	HTN
14	59	M	56	GN
15	69	F	34	DMN, HTN
16	71	F	58	GN
17	57	F	79	HTN
18	69	F	67	GN
19	34	M	35	DMN, HTN
20	72	F	105	Non-CKD
21	66	F	171	Non-CKD
22	59	M	106	Non-CKD
23	58	F	92	Non-CKD
24	69	M	79	Non-CKD
25	68	M	79	Non-CKD
26	69	F	76	Non-CKD
27	68	F	59	Non-CKD
28	52	M	75	Non-CKD
29	27	M	108	Non-CKD
30	29	F	108	GN
31	39	F	45	HTN
32	38	F	120	GN
33	43	M	25	HTN
34	40	M	23	HTN

35	65	F	77	HTN
36	64	M	104	GN
37	67	M	68	DMN
38	53	M	29	GN
39	73	M	15	DMN, HTN
40	52	M	57	GN
41	66	M	65	GN
42	76	M	33	GN
43	68	M	65	DMN
44	71	M	23	GN
45	72	M	53	DMN, HTN
46	63	M	41	HTN
47	45	M	41	GN
48	66	M	34	DMN, HTN
49	55	F	33	DMN, HTN
50	75	M	35	DMN, HTN
51	44	M	19	HTN
52	40	M	66	GN
53	59	M	60	DMN
54	53	M	56	HTN
55	49	M	57	HTN
56	73	F	87	Non-CKD
57	64	F	77	Non-CKD

[a] GN: glomerulonephritis; DMN: diabetic nephropathy; HTN: hypertension; CKD: chronic kidney disease.

Supplemental Table S3. Results of correlation analysis of natural abundances and normalized abundances of 26 IgG glycopeptides to the eGFR values.

**Glycopeptides**	**Natural abundance**		**Normalized abundance**	
	r	***p***	***r***	***p***
(1) H4N4F1-IgG1	0.4372	0.0008	0.1838	0.1752
(2) H5N4F1-IgG1	0.3844	0.0034	0.1104	0.4178
(3) H3N4F1-IgG1	0.2715	0.0430	0.1027	0.4513
(4) H4N4-IgG1	0.1823	0.1788	-0.0860	0.5293
(5) H5N4-IgG1	0.1155	0.3967	-0.1181	0.3861
(6) H4N5F1-IgG1	0.3191	0.0165	0.0667	0.6254
(7) H3N5F1-IgG1	0.2275	0.0918	-0.0028	0.9834
(8) H5N5F1-IgG1	0.3188	0.0166	0.0349	0.7982
(9) H4N5-IgG1	0.3892	0.0030	0.1504	0.2685
(10) H5N4F1S1-IgG1*	0.3926	0.0028	0.2501	0.0630
(11) H4N4F1S1-IgG1*	0.4538	0.0004	0.3727	0.0047
(12) H3N4F1-IgG3/4	0.1412	0.2991	-0.0835	0.5407
(13) H4N4F1-IgG3/4	0.2358	0.0802	0.1771	0.1916
(14) H3N5F1-IgG3/4	0.1434	0.2919	-0.1090	0.4239
(15) H4N5F1-IgG3/4	0.2601	0.0529	0.0466	0.7329
(16) H5N4F1S1-IgG3/4	0.2452	0.0686	0.0797	0.5593
(17) H5N4F1-IgG2	0.3248	0.0146	0.0206	0.8801
(18) H5N5F1-IgG2	0.2995	0.0250	0.0448	0.7433
(19) H4N4F1-IgG2	0.3510	0.0080	0.2104	0.1196
(20) H4N5F1-IgG2	0.3189	0.0166	0.0679	0.6192
(21) H4N4-IgG2	0.3394	0.0105	0.0373	0.7847
(22) H4N5-IgG2	0.3406	0.0102	0.1604	0.2376
(23) H3N4F1-IgG2	0.3246	0.0146	0.0853	0.5321
(24) H3N5F1-IgG2	0.3357	0.0114	0.0280	0.8377
(25) H5N4F1S1-IgG2	0.3636	0.0059	0.1290	0.3435
(26) H4N4F1S1-IgG2	0.3981	0.0024	0.1724	0.2038

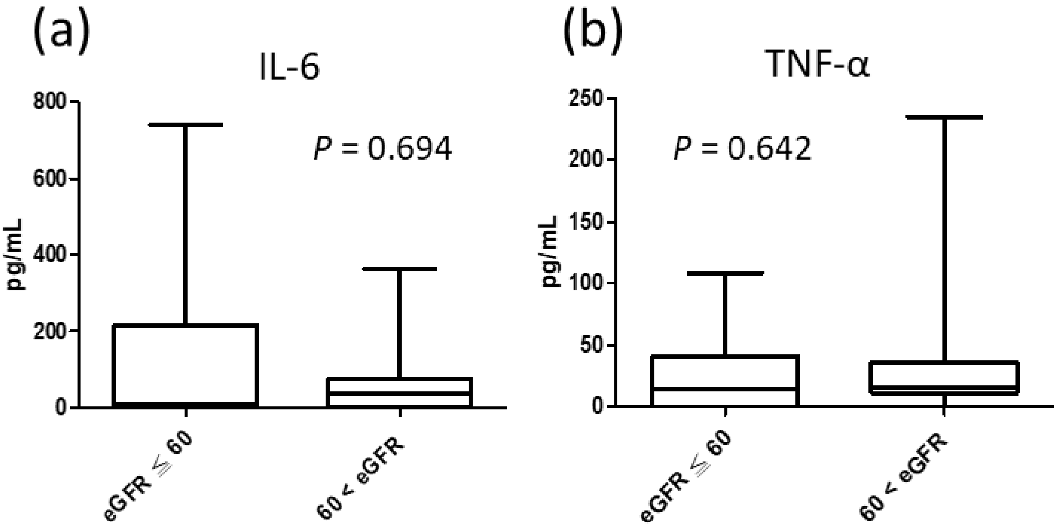

Supplemental Figure S1. Statistical analysis of plasma concentrations of IL-6 (a) and TNF-α (b) between groups with eGFR values lower and higher than 60 mL/ min/1.73 m^2.
